# Prevalence of Alcohol Use and Associated Factors in Urban Hospital Outpatients in South Africa

**DOI:** 10.3390/ijerph8072629

**Published:** 2011-06-28

**Authors:** Supa Pengpid, Karl Peltzer, Hendry Van der Heever

**Affiliations:** 1 Department of Health System Management and Policy, University of Limpopo, Ga-Rankuwa Campus, Medunsa, 0204, Pretoria, South Africa; E-Mail: Hendry_Heever@embanet.com; 2 HIV/AIDS/SIT/and TB (HAST), Human Sciences Research Council, 134 Pretorius Street, Pretoria, 0002, South Africa; E-Mail: Kpeltzer@hsrc.ac.za; 3 Department of Psychology, University of Limpopo, Turfloof Campus, Sovenga, 0727, Limpopo, South Africa

**Keywords:** alcohol misuse, tobacco use, associated factors, hospital out-patients, South Africa

## Abstract

The aim of this study was to assess the prevalence of alcohol use and associated factors among outpatients in an urban hospital in South Africa. The sample included 1,532 (56.4% men and women 43.6%) consecutively selected patients from different hospital outpatient departments. Results indicate that 41.2% of men and 18.3% of women were found to be hazardous drinkers, and 3.6% of men and 1.4% of women meet criteria for probable alcohol dependence or harmful drinking as defined by the Alcohol Use Disorder Identification Test (AUDIT). Two in five patients (40.5%) were hazardous or harmful drinkers and/or had anxiety or depression. Logistic multiple regression found that for men tobacco use and not having been diagnosed with diabetes and for women tobacco use and having been diagnosed with migraine headache was associated with hazardous and harmful drinking. Although the study is cross-sectional, it does identify groups that may be at high risk of alcohol misuse and for whom intervention is urgent. Because prevalence of hazardous and harmful alcohol use is high in this population, routine screening should be introduced in hospital out-patient settings.

## 1. Introduction

Hazardous drinking is defined as a quantity or pattern of alcohol consumption that places patients at risk for adverse health events, while harmful drinking is defined as alcohol consumption that results in adverse events (e.g., physical or psychological harm) [1,2]. Prevalence estimates range from 4% to 29% for hazardous drinking and from less than 1% to 10% for harmful drinking globally [1]. Similar prevalence rates of hazardous or harmful alcohol use in health care settings have been reported from different African countries [3–10].

Findings from the last nationally representative survey in South Africa including alcohol use found that risky or hazardous or harmful drinking was reported by 9%, 17% among men and 2.9% among women. Risky drinking was among men associated with 20 to 54 year age groups, the Coloured population group, lower economic status and lower education, and among women urban residence, the Coloured population group, lower education and higher income [11]. The South African population falls mainly into four racially defined social groups: black Africans, whites, coloureds (derived from Asian, European, and Khoisan and other African ancestry) and Asians (mainly Indians). Although defining social groups by race has its disadvantages as these groups do not have anthropological or scientific validity, it is useful because there are important differences between the groups for many indicators of health. These differences are mediated by cultural, political, and economic factors.

Previous studies found that anxiety or depression and tobacco use were associated with alcohol use disorders [12–15]. Anxiety and mood disorders are highly common among patients in primary care settings [16]. Identifying the alcohol use patterns in the subgroup of primary care patients diagnosed with anxiety and/or mood disorders may be particularly important [16].

Increasing emphasis has been placed on the detection and treatment of hazardous and harmful drinking disorders, particularly among patients who are seen in primary care settings [1]. Hospital settings are a particularly valuable point of contact for the delivery of brief interventions because of the large access to patient populations each year [17–19]. Little information exists about hazardous and harmful drinking disorders among hospital out-patients in South Africa which prompted the study.

## 2. Methodology

Sample and procedure: The sample included 1,532 hospital out-patients consecutively recruited from five different out-patient departments, *i.e.*, family practice (159, 10.4%), general out-patient department (735, 48.0%), cardiology (160, 10.5), diabetes (297, 19.4%) and ear nose and throat department (108, 7.1%) and from a dispensary (72, 4.7%). All out-patients were interviewed by four trained research assistants (qualified nursing assistants) in private rooms as they waited for their medical visit or at the dispensary throughout all hours of clinic operation for a period of three months in one tertiary hospital. The latter serves a large urban predominantly Black African population in Gauteng Province. Because of the stigma associated with alcohol consumption, individuals may feel defensive when responding to questions about their drinking and answer inaccurately. To increase the reliability of the AUDIT, researchers have suggested putting alcohol consumption in the context of other health-related behaviours [20,21]. Therefore, the interviewer administered questionnaire included questions on mental and physical health status, tobacco use and chronic diseases. The study protocol was approved by the Research Ethics Committee of The University of Limpopo, Medunsa Campus and the Hospital Chief Executive Officer. Informed consent was obtained from the participating patients. Patients were not paid for participation.

### 2.1. Measures

#### Demographic Characteristics

A researcher-designed questionnaire is used to record information on participants’ age, gender, educational level, marital status, income, and residential status.

#### Alcohol consumption

The 10-item Alcohol Disorder Identification Test (AUDIT) [22] assesses alcohol consumption level (three items), symptoms of alcohol dependence (three items), and problems associated with alcohol use (four items). Heavy episodic drinking is defined as the consumption of six standard drinks (10 g alcohol) or more on a single occasion [22]. In South Africa a standard drink is 12 g alcohol. Because AUDIT is reported to be less sensitive at identifying risk drinking in women [23], the cut-off points of binge drinking for women (four units) were reduced by one unit as compared with men (five units), as recommended by Freeborn *et al*. [23]. Responses to items on the AUDIT are rated on a 4-point Likert scale from 0 to 4, for a maximum score of 40 points. Higher AUDIT scores indicate more severe levels of risk; scores 8 or more indicate a tendency to problematic drinking. The Alcohol Use Disorders Identification Test (AUDIT) was developed by the World Health Organization as an effective screening instrument for alcohol use problems among patients seeking primary care for other medical problems in international settings including African countries (Kenya and Zimbabwe) [20,22] and has been widely used as standard tool in South Africa [8,11]. Cronbach alpha for the AUDIT in this sample was 0.88, indicating excellent reliability.

#### Tobacco use

Two questions were asked about the use of tobacco products. (1) Do you currently use one or more of the following tobacco products (cigarettes, snuff, chewing tobacco, cigars, *etc*.)? Response options were “yes” or “no”. (2) In the past month, how often have you used one or more of the following tobacco products (cigarettes, snuff, chewing tobacco, cigars, *etc*.)? Response options were once or twice, weekly, almost daily and daily.

#### Perceived general health

Participants were asked—In general, would you say your health is: excellent, very good, good, fair or poor? This measure was categorized based on participant response (very good = excellent/very good, good, and poor = fair/poor).

The *Kessler Psychological Distress Scale* (K-10): was used to measure global psychological distress, including significant pathology which does not meet formal criteria for a psychiatric illness [24,25]. This scale measures the following symptoms over the preceding 30 days by asking: “In the past 30 days, how often did you feel: nervous; so nervous that nothing could calm you down; hopeless; restless or fidgety; so restless that you could not sit still; depressed; that everything was an effort; so sad that nothing could cheer you up; worthless; tired out for no good reason?” The frequency with which each of these items was experienced was recorded using a five-point likert scale ranging from “none of the time” to “all the time”. This score was then summed with increasing scores reflecting an increasing degree of psychological distress. The K-10 has been shown to capture variability related to non-specific depression, anxiety and substance abuse, but does not measure suicidality or psychoses [26]. This scale serves to identify individuals who are likely to meet formal definitions for anxiety and/or depressive disorders, as well as to identify individuals with sub-clinical illness who may not meet formal definitions for a specific disorder [24]. This scale is increasingly used in population mental health research and has been validated in multiple settings [27] including among pregnant women [28] and HIV positive individuals in South Africa [29]. We examined the K-10 scale using ordinal categories for low, moderate, high and very high psychological distress (scores of 10–19, 20–24, 25–29 and 30 or more, respectively) and as a binary variable comparing scores of 0–29 versus 30 or more. The internal reliability coefficient for the K-10 in this study was alpha = 0.89.

Patients were also asked about a list of chronic and other illness conditions they had been diagnosed with such as hypertension, diabetes and sexually transmitted diseases.

### 2.2. Data Analysis

Data were analyzed using Statistical Package for the Social Sciences (SPSS) for Windows software application programme version 18.0. Frequencies, means, standard deviations, were calculated to describe the sample. Data were checked for normality distribution and outliers. For non-normal distribution non-parametric tests were used. Associations of hazardous or harmful alcohol use were identified using logistic regression analyses. Following each univariate regression, multivariable regression models were constructed. Independent variables from the univariate analyses were entered into the multivariable model if significant at P < 0.05 level. Logistic regression was conducted for men and for women separately. Cases with missing data were excluded from the multivariable models. The rate of missing data never exceeded 5% of cases. Excluded cases differed from included cases in terms of older age (P < 0.05). For each model, the R^2^ are presented to describe the amount of variance explained by the multivariable model. Probability below 0.05 was regarded as statistically significant.

## 3. Results and Discussion

### 3.1. Sample Characteristics

From the 1,713 approached hospital out-patients 1,532 agreed to participate (89.4% response rate). The final sample included 1,532 (56.4% men and women 43.6%), the mean age was 36.1 years (SD = 11.6), range 18 to 77 years. Almost all (99.4%) belonged to the Black African population group, while 0.6% were Coloured or White. Majority (60%) were general and 40% chronic hospital outpatients, 24.2% used tobacco products daily or almost daily, and 17.1% scored on the psychological distress scale 30 or more indicating severe psychological distress (see Table 1).

### 3.2. Alcohol Use and Co-Morbidity

Using a cut-off score of 8 to 19 for the AUDIT analysis indicated that 41.2% of all men and 18.3% of all women were classified as hazardous drinkers, and 5.3% of men and 1.4% of women meet criteria for probable alcohol dependence (harmful drinking) (with an AUDIT score of 20 or more) as defined by AUDIT. Men had significantly higher AUDIT scores than women (see Table 2).

### 3.3. Associations of Hazardous or Harmful Alcohol Use

Univariate analyses found that among men younger age, tobacco use, psychological distress, migraine headache, not having had a heart attack or angina and not having diabetes were associated with hazardous or harmful alcohol use, while among women family contributions as a main household income, tobacco use, migraine headache and having been diagnosed with a stomach ulcer were associated with hazardous or harmful alcohol use. In multivariable analyses it was found that among men tobacco use and not having diabetes were associated with hazardous or harmful alcohol use, and among women that tobacco use and migraine headache were associated with hazardous or harmful alcohol use (see Table 3).

### 3.4. Discussion

Our results found high rates of hazardous or harmful drinking (34.8%) among hospital out-patients in South Africa. Previous studies using the same alcohol measure (the AUDIT = Alcohol Use Disorder Identification Test) in non-hospital settings found lower rates of hazardous or harmful alcohol use including in primary care in South Africa 13.3% [7] and 19.2% [8]; Nigeria 28.6% [4], in Harare, Zimbabwe (25%) in 2003 [5] follow by 18.3% in 2008 [9], in Sweden (11.9%) [30], and population surveys in South Africa (9%) [10]. Further, the study found that 40.5% were hazardous or harmful drinkers and/or had anxiety or depression, which was also higher than in previous studies in primary care, e.g., 31.9% in Sweden [30].

The high prevalence of hazardous or harmful alcohol use among men compared to women in this study as well as other studies in South Africa [8] may be explained by urbanization [9,11,31] and the low prevalence among Black African women may be due to cultural proscriptions against substance use [32]. Our other finding was that higher psychological distress was associated with increased risk for alcohol misuse. It may be that interventions to reduce psychological distress and to improve coping, are particularly important [9]. The implication of this found high prevalence of hazardous or harmful alcohol use in hospital out-patients compared to lower prevalence rates found primary care settings in South Africa seems to suggest that screening and brief intervention of alcohol problems should focus on hospital patients.

Factors associated with hazardous or harmful drinking in this study included male gender, tobacco use, psychological distress, migraine headache and not having diabetes. Other studies also found that male gender [9], employment [9] higher income [11], tobacco use [14], migraine headache [33] were associated with higher hazardous or harmful drinking. In terms of diabetes, contrary to this study, other studies found in men, both low and high alcohol consumption was associated with increased risks of having diabetes and in women alcohol consumption was associated with a decreased risk of having diabetes mellitus [34]. It is possible that diabetes patients after they have been diagnosed with diabetes reduce their alcohol consumption or diabetes patients in South Africa are less risky drinkers than among other patients. This is confirmed from national population based surveys in South Africa (unpublished data). The finding that migraine headaches were associated with hazardous or harmful use in this sample could indicate the possibility of alcohol use as trigger of migraine headaches. However, prospective studies limit considerably the importance of alcohol as a trigger [34].

Findings may indicate that screening for risky alcohol use should be integrated into routine hospital out-patient care. A feasible and attractive option is integrating the intervention into a broader lifestyle intervention [18].

### 3.5. Study Limitations

Caution should be taken when interpreting the results of this study because of certain limitations. As this was a cross-sectional study, causality between the compared variables cannot be concluded. A further limitation was that all variables were assessed by self-report and desirable responses may have been given. Other examples of limitations include: no drug screening, no standardized psychiatric measures (such as the Structured Interview for DSM-IV Axis I disorders). Further, we relied on self-report data; thus the prevalence rates are subject to biases such as problems recalling alcohol use and social desirability of responses [35]. However, the AUDIT has been found to be reliable and acceptable for screening use internationally [36], and to have validity similar to other established self-report instruments [37]. In addition, the interviewers were qualified nursing assistants wearing a uniform which may have led to more willingness to reveal health and lifestyle information.

## 4. Conclusions

The high prevalence of hazardous drinking found in the study highlights the need for brief intervention programmes with respect to hospital outpatients in South Africa. Treatment of these disorders in the form of brief interventions can be successfully accomplished in hospital settings, as demonstrated by a number of well-conducted randomized trials [17]. Future research can best be focused on the effectiveness of brief alcohol interventions among hospital outpatients in routine practice [18] and should seek to elucidate the mechanisms by which particular risk and protective factors such as urbanization and other factors operate [9].

## Figures and Tables

**Table 1:** Sample characteristics.

	Total (n = 1,532)		Men (n = 864) (56.4%)		Women (n = 668) (43.6%)			
	N or M	% or SD	N or M	% or SD	N or M	% or SD	χ^2^ or t	P
***Age***	36.1	11.6	37.2	12.3	34.7	10.5	−4.24	0.000
18–24	258	16.9	138	16.0	120	18.1		
25–34	528	34.6	280	32.6	248	37.3		
35–44	369	24.2	200	23.3	169	25.5		
45–54	247	16.2	153	17.8	94	14.2		
55 or more	122	8.0	89	10.3	33	5.0		
***Marital status***								
Never married	955	63.8	520	61.8	435	66.2	3.06	0.080
Married/cohabitating	472	30.5	289	34.3	183	27.8	7.24	0.007
Separated/divorced/widowed	58	5.7	32	3.8	29	5.9	3.71	0.054
***Education***								
Grade 7 or less	207	13.6	125	14.6	82	12.4	1.56	0.221
Grade 8–11	584	38.4	335	39.0	249	37.6	0.33	0.566
Grade 12 or more	731	48.0	399	46.4	332	50.1	1.97	0.160
***Main household income***								
Formal salary	497	32.6	325	37.8	172	25.9	23.24	0.000
Family member contributions	485	31.8	235	27.4	250	37.7	18.28	0.000
Social grants	144	9.5	68	7.9	76	11.4	5.45	0.020
Other	191	12.5	128	14.9	63	9.5	10.01	0.002
No income	206	15.5	103	12.0	103	15.5	3.39	0.042
Urban residence	1218	80.1	693	80.8	523	79.1	0.62	0.426
Rural residence	303	19.9	165	19.2	138	20.9		
Chronichospital out-patient	615	40.3	304	35.3	311	46.8	20.32	0.000
General hospital out-patient	910	59.7	556	64.7	354	53.2		
Very good vs. poor health status	882	57.8	498	58.0	383	57.6	0.02	0.881
Daily or almost daily tobacco use	370	24.2	288	33.4	82	12.3	91.11	0.000
Severe psychological distress (based on Kessler 10)	228	15.1	118	13.8	110	16.7	3.14	0.002
***Illness conditions (ever diagnosed)***								
Hypertension	293	19.3	148	17.2	145	21.9	5.27	0.022
High cholesterol	44	2.9	21	2.5	23	3.5	1.40	0.238
Diabetes	126	8.3	74	8.6	52	7.9	0.28	0.597
Cancer	48	3.2	17	2.0	31	4.7	8.91	0.003
Depression	107	7.0	57	6.7	50	7.6	0.48	0.490
Migraine headache	459	30.2	201	23.5	258	39.2	43.48	0.000
Stomach ulcer	263	17.3	125	14.6	138	21.0	10.68	0.000
Asthma	69	4.6	29	3.4	40	6.1	6.08	0.014
Arthritis	262	17.3	125	14.6	136	20.6	9.42	0.002
Tuberculosis	111	7.3	74	8.6	37	5.6	5.08	0.024
Lower back pain	394	26.1	207	24.4	187	28.4	3.12	0.078
Sexually transmitted disease (STD)	115	7.6	70	8.2	45	6.8	0.99	0.318

**Table 2:** Alcohol use and co-morbidity by sex in percent.

	AUDIT score	Total (n = 1,532)	Men (n = 864)	Women (n = 668)	χ^2^ or *	P
Abstainers	0	50.2	39.6	63.8	87.56	0.000
Low-risk drinkers	1–7	15.0	13.8	16.5	2.18	0.140
High risk drinkers	8–19	31.2	41.2	18.3	91.45	0.000
Probable alcohol dependence	20+	3.6	5.3	1.4	17.19	0.000
Hazardous or harmful drinkers	8+	34.8	46.5	19.7	119.33	0.000
Hazardous or harmful drinkers and severe psychological distress		16.6	17.1	14.8	14.97	0.000
Hazardous or harmful drinkers and (almost) daily tobacco products user		45.6	51.1	29.0	91.77	0.000
Hazardous or harmful drinkers and/or severe psychological distress		40.5	47.9	31.3	40.98	0.000
		M (SD)	M (SD)	M (SD)		
Total AUDIT score		5.3 (6.7)	7.1 (7.3)	3.1 (5.1)	*	0.000

* *Mann-Whitney U test* = P < 0.001.

**Table 3:** Associations of hazardous or harmful alcohol use.

	Men		Women	

	Cr OR (95% CI) a	Adj OR (95% CI) a,b	Cr OR (95% CI) a	Adj OR (95% CI) a,c

Age	0.99 (0.98–1.00) *	0.99 (0.98–1.00)	0.98 (0.97–1.00)	---

Married/cohabitating *vs.* Not	0.78 (0.59–1.04)	---	0.80 (0.52–1.25)	---

Education	1.03 (0.97–1.10)	---	0.99 (0.90–1.08)	---

No income-Ref	---	---	---	---
Formal salary	0.92 (0.59–1.43)		1.04 (0.59–1.83)	1.37 (0.74–2.54)
Family contributions	0.77 (0.48–1.22)		0.56 (0.32–0.99) *	0.82 (0.44–1.56)
Social grants	0.65 (0.35–1.20)		0.64 (0.31–1.36)	0.86 (0.38–1.94)
Other	0.89 (0.53–1.50)		0.73 (0.34–1.59)	1.01 (0.44–2.31)

Urban vs. Rural residence	0.93 (0.67–1.31)	---	1.00 (0.62–1.60)	---

Chronic *vs.* General out-patient	1.03 (0.78–1.36)	---	1.34 (0.92–1.97)	---

Very good *vs.* Poor health status	1.10 (0.84–1.45)	---	0.91 (0.62–1.34)	---

Daily or almost daily tobacco use	4.75 (3.49–6.46) ***	4.59 (3.30–6.39) ***	4.55 (2.80–7.41) ***	4.38 (2.65–7.27) **

Psychological distress score	1.02 (1.00–1.04) **	1.01 (0.99–1.03)	1.00 (0.89–1.02)	---

Depression diagnosed	1.20 (0.70–2.05)	---	0.88 (0.42–1.86)	---

Heart attack or angina diagnosed	0.59 (0.40–0.87) **	1.47 (0.93–2.33)	0.85 (0.48–1.49)	---

Hypertension	0.74 (0.52–1.06)	---	0.96 (0.60–1.53)	---

STD	1.48 (0.91–2.42)	---	0.73 (0.32–1.68)	---

Ulcer	1.11 (0.76–1.62)	---	1.60 (1.03–2.48) *	1.18 (0.72–1.95)

Migraine headache	1.49 (1.08–2.04) *	1.36 (0.92–2.06)	1.78 (1.21–2.61) **	1.61 (1.07–2.43) *

Asthma	1.23 (0.59–2.58)	---	1.58 (0.77–3.25)	---

High cholesterol	0.45 (0.17–1.17)	---	0.38 (0.09–1.62)	---

Diabetes	0.49 (0.29–0.81) **	0.56 (0.31–0.99) *	0.50 (0.21–1.21)	---

Arthritis	1.19 (0.81–1.74)	---	1.00 (0.62–1.61)	---

Bronchitis	0.94 (0.51–1.76)	---	1.05 (0.45–2.47)	---

Tuberculosis	1.23 (0.76–1.98)	---	1.12 (0.50–2.52)	---

Cancer	0.35 (0.11–1.02)	---	0.97 (0.39–2.41)	---

a Using “enter” LR selection of variables;

b For men Hosmer and Lemeshow Chi-square 7.01, df 6, 0.536; Cox and Snell R^2^ 0.13; Nagelkerke R^2^ 0.21;

c For women Hosmer and Lemeshow Chi-square 11.91, df 6, 0.064; Cox and Snell R^2^ 0.15; Nagelkerke R^2^ = 0.22;

* P < 0.05;

** P < 0.01;

*** P < 0.001.
